# Crafting Jobs for Occupational Satisfaction and Innovation among Manufacturing Workers Facing the COVID-19 Crisis

**DOI:** 10.3390/ijerph17113953

**Published:** 2020-06-03

**Authors:** Tianzhou Ren, Lele Cao, Tachia Chin

**Affiliations:** School of Management, Zhejiang University of Technology, Hangzhou 310023, China; rentianzhou@zjut.edu.cn (T.R.); caolele1028@163.com (L.C.)

**Keywords:** COVID-19, job crafting, occupational satisfaction, innovation, China

## Abstract

China’s manufacturing employees are confronted with unprecedent occupational and innovation challenges caused by the ongoing COVID-19 crisis coupled with the pressure of being replaced by digital technologies. To gain a better understanding of the rising occupational uncertainty during this critical time, based on the job demands-resources (JD-R) theory, we examined the associations of employees’ job crafting behaviors (JCB) with their occupational satisfaction and innovation workplace behavior (IWB), as well as the mediating effect of work engagement on the above relationships. The final usable data were obtained from the formal survey of 311 employees of six manufacturing companies that have returned to work amid COVID-19. Structural equation modelling was adopted to analyze the data. Results show that employees’ JCB strengthens their occupational satisfaction and IWB via work engagement. Theoretically, our research enriches the existing body of knowledge about JCB from a cross-disciplinary angle integrating the perspectives of career and psychology. Practically, we offer valuable first-hand evidence about how manufacturing employees conducted JCB to re-orient their careers and to innovate in the face of the high unemployment situation.

## 1. Introduction

The sudden outbreak of COVID-19 has caused an unprecedent global recession, which is crunching the job market in China’s manufacturing industry. It is primarily due to the fact that the increasing economic volatility and psychological insecurity about human coronavirus infection is resulting in many factories, particularly those export-oriented, shedding workers and putting more dependence on information and communication technologies (ICTs), such as smart machines and artificial intelligence (AI) to reduce the number of human workers. In this vein, production workers cannot confront the increasingly complex, more ICT-related job demands, while the low-skilled, less-educated and middle-aged workers who used to engage in simple, repetitive tasks in the past, are becoming less competitive and experiencing more job stress. As a result, in such a turbulent time, manufacturing employees may gradually lose their occupational satisfaction, as the success and progress they have made in their careers may not be sustainable. Moreover, it implies the need of developing and encouraging innovative workplace behavior (IWB) among factory workers [1,2,3] to cope with such a tough situation.

According to the job demands-resources (JD-R) theory [4], there are two general categories of job characteristics for common occupations: job demands and job resources. Some proactive workers tend to satisfy the rising job demands by undertaking job crafting behaviors (JCBs) [5,6,7,8], namely to take the initiatives to acquire new job-related resources, re-configure the resources within their jobs, or decrease hindering demands, with an aim to better align job characteristics with their own preferences and needs. The recent, ongoing market turbulence triggered by the COVID-19 pandemic, coupled with the pressure of digitalization, has largely increased the job demands in manufacturing and production workers. For instance, given the coronavirus is transmitted by close contact with an infected person, quite a few manufacturing firms have deployed robots to replace human workers to disinfect plants and provided ICT-facilitated customer services. In this vein, workers may opt to conduct JCBs for reaching a new balance of job resources and demands, so as to ameliorate their occupational uncertainty, realize innovation, and thereby achieve competitive advantages [7,8,9,10]. Following this line of thought, we focus on exploring the associations among employee JCB, occupational satisfaction, and IWB here.

It should be noted, the pivotal role of JCB has been widely recognized in the domain of organizational psychology [7], and several studies have demonstrated the significant relationships of employee JCB to IWB and other positive behaviors [9,11]. However, hitherto there have been limited research addressing the impacts of JCB on IWB and occupational satisfaction among manufacturing workers, particularly in non-western contexts [12], or from a cross-disciplinary perspective [13]. Taken together, this paper thus aims to fill the foregoing knowledge void by investigating how JCB affects occupational satisfaction and IWB among China’s manufacturing workers during COVID outbreak, from an integrative perspective of career and psychology. Given work engagement as a critical psychological resource has been discovered to mediate the association between JCB and work performance [10], we also examine the mediating role of work engagement here.

## 2. Literature Review and Hypothesis Development

### 2.1. The Expansive Dimensions of JCB

The notion of job crafting (JC) was first conceptualized by Wrzesniewski and Dutton as “the physical and cognitive changes individuals make in their task or relational boundaries in order to make their job more meaningful” [14]. Extending their line of thought, but expurgating the aspect of cognitive crafting, Tims, Bakker, and Derks [15] applied the JD-R theory, where job demands refers to the aspects of the job that require energy and efforts at work, and job resources as the job aspects that provide motivation and energy to employees at work into interpreting the notion of JC; they further defined JC as the changes a person makes to better fit the balance between job demands and job resources in accordance with their personal needs and abilities. They then empirically categorized JC into the following four dimensions: (1) increasing structural job resources (e.g., incorporating task autonomy, task variety, and opportunities for development), (2) increasing social job resources (e.g., crafting feedback from their colleagues), (3) increasing challenging job demands (e.g., new projects), and (4) decreasing hindering job demands (making works mentally less intense). Due to its good operationality, the four-dimension JC scale proposed by Tims et al. [15] has been frequently adopted in quantitative studies, while Wrzsniewski and Dutton’s [14] (including the perspective of cognitive crafting) was mostly applied in qualitative studies [6]. Given the empirical nature, we followed Tims et al.’s [15] definition of JC here.

According to the literature [16,17,18], the first three dimensions of JC were regarded as the “expansive” JC as they characterize the increasing of social and structural resources, and challenging demands, and often promote positive work behaviors and outcomes, while the fourth dimension “decreasing hindrance job demands” appeared to be relatively “unexpansive” and prevention-oriented due to its negative associations with job satisfaction and performance. Rudolph, Katz, Lavigne, and Zacher [19] meta-analytically examined the construct validity of the four dimensions of JC, discovering that the factor loading of the hindering JC dimension was less than 0.05, which was much lower than the other three dimensions. Echoing this point of view, some empirical studies only discuss the expansive dimensions of the JC measure [18,20]. Scholars also claimed that the expansive JC activities seem to be much better fit for the motivational process of the JD-R model than the reducing JC activities [20]. Taking together the foregoing discussion, given the JD-R perspective used as our theoretical grounding, we thus only focus on the expansive JCB here.

It should be noted, considering our research background, an intriguing question is whether low skilled production workers are able to craft their jobs. Fortunately, several scholars have clearly answered this question by describing several examples of how low-skilled employees with less autonomous jobs, such as hairdressers and salespeople, crafted their jobs [14,15]. Their claims indicate that every employee can engage in some of JCB. In terms of frontline production workers, they may craft their jobs by proactively engaging in continuous improvement projects to enhance manufacturing efficiency and quality.

### 2.2. JCB and Occupational Satisfaction

Occupational satisfaction is defined as a person’s satisfaction towards his/her career accomplishments, such as the overall success, the goal progress, as well as the income and skill development [21], while the assessment of occupational satisfaction was built upon the nature of the match between the job design and individual’s occupational growth needs [22]. As noted at the outset, in China’s manufacturing industry, occupational satisfaction among factory workers has been largely ignored in the past [5], however it has come to the surface and become an imperative issue needing attention. Under the pressure of losing jobs, some proactive front-line workers may tend to perform JCB, whereby they are able to be more active and responsive to the environmental turbulence through increasing their social and structural resources, and thereby to feel more satisfied with their careers. Nevertheless, although JCB has been a hot topic in the field of psychology and human resource management, there is a prominent knowledge void linking JC to the career domain.

As per the JD-R theory [4], the conduct of expansive JCBs that involves leveraging and enriching a variety of job resources and challenging job demands is expected to promote individuals’ positive behaviors and psychological well-being at workplace [23,24]. Whereas occupational satisfaction represents a positive emotional status or a sense of happiness [21,22,25,26,27], we assume the following hypothesis against the backdrop of COVID-19 and digitalization:

**Hypothesis** **1a** **(H1a).** *Manufacturing employees’ expansive JCB is positively related to their occupational satisfaction.*

### 2.3. JCB and IWB

Prior literature has offered a variety of scales to measure employee innovation performance. Among them, Vegt and Janssen’s [28] conceptualization is a more comprehensive and widely recognized measure, in which IWB is constituted by three dimensions: the intentional generation, promotion, and realization of novel and useful ideas within a job, a group, or an organization. Because this IWB measure involves much broader and intricate meaning than individual creativity, we adopted it to evaluate employees’ IWB here. It is worth noting that when enhancing employee IWB, how to mobilize the innovation potential of the employees with valuable tacit knowledge has been a crucial task. It is because, as far as the externalization of tacit knowledge is concerned, the intrinsic-driven motivators (e.g., job resources and challenges) are more catalytic than extrinsic-driven motivators (i.e., incentives and compensations) [29].

JC is deemed as a critical intrinsic-driven motivator and has been found to promote a variety of employees’ positive job outcomes, such as organizational citizenship behaviors and task performance [19]. Hence, it is plausible that increasing structural job resources will help employees craft innovative thoughts, increasing social job resources will help nurture new ideas, products, and processes in organizations, and increasing challenging job demands will help spur overall organizational innovation engagement. Therefore we hypothesize:

**Hypothesis** **1b** **(H1b).** *Manufacturing employees’ expansive JCB is positively related to their IWB.*

### 2.4. JCB and Work Engagement

Quite a few scholars have claimed that work engagement—defined as a positive, fulfilling, work-related state of mind, including vigor, dedication and absorption dimensions [30]—can be strengthened by the expansive JCB [10,15,31]. For instance, in a study of flight attendant employees working at an Iranian airline company [32], it was discovered that when flight attendants had the option to craft their jobs through increasing job resources and challenging job demands (i.e., carrying out expansive JCB), they felt enthusiastic and inspired by their job. Cullinane et al. [33] conducted a study in the European pharmaceutical division, indicating that the expansive JCB is an antecedent to employee engagement behaviors. Rudolph et al. [19] also indicated that the expansive JCB is positively associated with work engagement.

In light of the JD-R theory, facing rising job stress in current tough employment situation, workers with better resources are very likely to proactively change their work atmosphere and thereby modify their jobs to become more challenging and resourceful (i.e., engaging in expansive JCB). In this vein, their engagement at work is expected to be promoted. Following this logic, we hypothesize:

**Hypothesis** **2** **(H2).** *Manufacturing employees’ expansive JCB is positively related to their work engagement.*

### 2.5. The Association of Work Engagement with Occupational Satisfaction and IWB

The positive association of work engagement with extra-role or proactive behaviors has been widely identified [34,35]. In terms of IWB, García-Buades et al. [36] have provided collective-level findings showing that engaged employees usually ‘infect’ their colleagues with their enthusiasm and vigor as an emotional contagion, whereby all their co-workers are more willing to perform IWB because they perceive a supportive and passionate innovative climate. Moreover, work engagement has been found to be a vital antecedent of individuals’ job satisfaction and subjective occupational success [37,38,39].

As per the JD-R framework, under tough work conditions, engaged employees who often experience more positive emotions seem more eager to implement altruistic behaviors that benefit their organizations, which in turn also helps sustain their careers. Chinese manufacturing workers are striving for more job resources to tackle the high unemployment risk coupled with increasingly complex job demands during this critical period, therefore we predict that engaged workers may be more inclined to perform IWB and feel more satisfied with their careers:

**Hypothesis** **3a** **(H3a).** *Manufacturing employees’ work engagement is positively related to their IWB.*

**Hypothesis** **3b** **(H3b).** *Manufacturing employees’ work engagement is positively related to their occupational satisfaction.*

### 2.6. The Mediating Role of Work Engagement

Recent studies have also reported that employees’ work engagement may exert a crucial intervening effect on the job characteristics–performance relationships [35], on the personal resources–work performance associations [9], as well as on the links of JC with extra-role behaviors [8] and with job performance [40]. These arguments seem to implicitly indicate a mediating role that work engagement may play in affecting between employee JC and IWB.

Our Hypotheses 1a, 1b, and 2 have proposed the positive associations of JC to occupational satisfaction, IWB and work engagement, while Hypotheses 3a and 3b have suggested the positive relations from work engagement to occupational satisfaction and IWB. Taking together, it seems plausible to predict a continuum of JC-work engagement–occupational satisfaction/IWB. In this vein, we further hypothesize the following:

**Hypothesis** **4a** **(H4a).** *Manufacturing employees’ work engagement mediates the JC–occupational satisfaction relationship.*

**Hypothesis** **4b** **(H4b).** *Manufacturing employees’ work engagement mediates the JC–IWB relationship.*

### 2.7. Age as the Boundary Condition of our Proposed Mediated Model

According to the JD-R theory, the sudden outbreak of COVID-19 caused rising job demands, which has resulted in an increasing number of manufacturing employees seeking a variety of job resources to eliminate the costs of demands. It is thus plausible to see the frequent happening of organizational changes amid this crisis. Quite a few studies report that engaging in JCB can be seen a response or reaction of employees to such organizational changes, where employee age often acts as a critical control variable affecting the relationship of JCB with individual job- and career-related outcomes [5,7,8,18,19,20,21]. For instance, older employees are usually found to be less innovative and less motivated amid high uncertainty and be more associated with the stereotype of being more resistant to change [3,5,22]. Following this logic, we further argue that manufacturing workers’ age is very likely to serve as a boundary condition for our proposed model and posit:

**Hypothesis** **5a** **(H5a).** *Manufacturing employees’ age moderates the above-mentioned mediating model, such that the mediating effect of work engagement on the JC–occupational satisfaction relationship is stronger among younger employees.*

**Hypothesis** **5b** **(H5b).** *Manufacturing employees’ age moderates the above-mentioned mediating model, such that the mediating effect of work engagement on the JC–IWB relationship is stronger among younger employees.*

## 3. Method

### 3.1. Participants

Whereas it was difficult to move between provinces amid the COVID-19 outbreak, we chose six clothing manufacturers located in Zhejiang province as our sample firms. This research project was initiated in late March 2020 and lasted for 25 days including two time points. In each firm, we had at least one contact person from the human resource (HR) department to help us distribute online questionnaires. Before the formal surveys, we carried out in-depth telephone interviews with several HR professionals and experienced production workers from the selected firms to ensure the properness and clearness of our measurements and hypotheses. Their feedback showed that our hypothesized framework was logical. Moreover, we also asked them what HR strategy was conducted when their factories were temporarily closed amid the COVID-19. All six firms claimed that they had laid off some workers for saving costs because they didn’t know when the market would recover and when migrant workers would be allowed to come back to work due to the human mobility restrictions; two export-oriented firms even mentioned that they were thinking about closing the factories permanently owing to the imposed COVID-19-related export constraints. To ensure the validity of our research, we only selected the workers who were still employed as our participants. In terms of the formal online survey, we used the well-known online service provider in China, namely the sojump.com (https://www.wjx.cn/register/register.aspx?type=1) to create and distribute our questionnaires to participating workers via Wechat. We also provided a clear guideline about how survey participants should fill out the questionnaire and how their anonymity was protected. In addition, a Wechat account/ID was offered to answer the inquiries from participants.

According to the demographic structure and description (e.g., workers’ age and gender distribution) of each factory, we randomly chose 450 production workers from the six firms’ staff name lists. To reduce extraneous sources of variation and measurement error, we excluded part-time and foreign workers. With the help from HR personnel, data were collected at two time points as the time-lag research design can reduce the likelihood of common method variance (CMV) [41]. We measured JC at Time 1, and work engagement, IWB, and career satisfaction at Time 2 (20 days later). As a result, usable data from a total of 307 employees (with both Time 1 and Time 2 data) were obtained, with a response rate of 68.8%. The sample included 228 females and 79 males, with an average age of 37 years (standard deviation (SD) = 8.24) and an organizational tenure of 10 years.

### 3.2. Measures

All the questionnaires were originally developed in English but were administered in Chinese in light of the translation procedure [42]. Additionally, the questionnaires were emailed to the managers of each organization to ensure that our items were easily understood and made sense within the firm. The participants were asked to respond on a 6-point Likert-type scale ranging from (1) “strongly disagree” to (6) “extremely agree”, so as to prevent response bias because Chinese employees are apt to choose the midpoint of the scale [12,43].

JC was measured with the Tims et al. (2012) [15] 15-item scale. The same scale has been widely adopted in management studies [44]. Sample items include the following: ‘I try to learn new things at work’, ‘I look to my supervisor for inspiration’, and ‘I try to make my work more challenging by examining the underlying relationships between aspects of my job’, etc. (Cronbach’s α = 0.977).

Work engagement was measured with the Schaufeli, Bakker, and Salanova [45] 9-item scale. These items have been widely employed to assess individual work engagement in the literature and demonstrated good reliability [16]. Sample items include ‘At my job, I feel strong and vigorous’ (vigor), ‘My job inspires me’ (dedication), and ‘I am immersed in my work’ (absorption; Cronbach’s α = 0.968).

To assess occupational satisfaction, the 5-item scale developed by Greenhaus, Parasuraman, and Wormley [22] was used; a sample item is ‘I am satisfied with the success I have achieved in my career’ (Cronbach’s α = 0.938).

We measured individual-level IWB using the scale developed by Vegt and Janssen [28]. The employees were asked to rate their self-innovative behaviors. Sample items from this scale included ‘Searching out new working methods, techniques, or instruments’, ‘Acquiring approval for innovative ideas’, and ‘Introducing innovative ideas into the work environment in a systematic way’ (Cronbach’s α = 0.961).

The statistical models controlled for three additional variables: gender (0 = male; 1 = female), age (in years), and experience (in years).

### 3.3. Ethics Statement

This study was conducted in accordance with the ethical guidelines of the Institutional Review Board of Zhejiang University of Technology (ZJUT) in China, with written informed consent from all subjects. All the employees participated in the survey voluntarily. The protocol was approved by the Institutional Review Board of ZJUT and the Secretariat of Academic Committee of ZJUT, with the permit number 2020004.

## 4. Results

### 4.1. Confirmatory Factor Analysis and Descriptive Statistics

Before testing our hypotheses, we assessed the discriminant validity of our measurement model using a series of confirmatory factor analyses (CFAs) with AMOS24.0 (IBM, Armonk, NY, USA). The fit of the proposed four-factor model was adequate (χ^2^ = 1602.130, df = 540, RMR = 0.022, RMSEA = 0.08, CFI = 0.932). We also examined another five alternative models: a three-factor model (Model A) combining JC and work engagement (χ^2^ = 2509.446, df = 547, RMR = 0.049, RMSEA = 0.108, CFI = 0.874), a three-factor model (Model B) combining IWB and occupational satisfaction (χ^2^ = 2232.142, df = 547, RMR = 0.03, RMSEA = 0.10, CFI = 0.892), a two-factor model (Model C) combining JC, work engagement, and IWB (χ^2^ = 3278.853, df = 549, RMR = 0.046, RMSEA = 0.127, CFI = 0.825), a two-factor model (Model D) combining job crafting, work engagement, and occupational satisfaction (χ^2^ = 2966.695, df = 549, RMR = 0.044, RMSEA = 0.119, CFI = 0.845), and a one-factor model (Model E) combining job crafting, work engagement, IWB, and occupational satisfaction (χ^2^ = 3626,738, df = 550, RMR = 0.046, RMSEA = 0.134, CFI = 0.803). Comparing the fit indices of our proposed model with the five alternative models, it is clear that the proposed four-factor construct showed a better model fit and exhibited adequate discriminant validity. (Note: df = degrees of freedom; RMR = Root Mean Square Residual; RMSEA = Root Mean Square Error of Approximation; CFI = Comparative Fit Index).

Table 1 summarizes the correlations, descriptive statistics, and internal consistency of the research variables. The correlation results revealed that JC is positively associated with occupational satisfaction (r = 0.725, *p* < 0.01), IWB (r = 0.719, *p* < 0.01), and work engagement (r = 0.658, *p* < 0.01), and work engagement is positively related to IWB (r = 0.707, *p* < 0.01) and occupational satisfaction (r = 0.712, *p* < 0.01). These results are consistent with the direction of our hypotheses, thus offering preliminary evidence for the validation of the hypotheses.

### 4.2. Testing of Hypotheses

We performed structural equation modelling (SEM) to test our hypotheses. Moreover, we followed the procedures suggested by MacKinnon, Lockwood, Hoffman, West, and Sheets [46] to build a mediation model. To confirm the mediation associations, three sets of SEM (Structural Equation Modelling) models with all three controlled variables added were computed. More specifically, we chose the full mediation model (JC→Work engagement→Occupational satisfaction and IWB) as our baseline model and compared it with a partial mediation model (including the direct relationships from job crafting to occupational satisfaction and IWB to the baseline model) and the non-mediation model (reducing the mediating relationships from JC to work engagement and from work engagement to occupational satisfaction and IWB from the baseline model).

**Figure 1:**
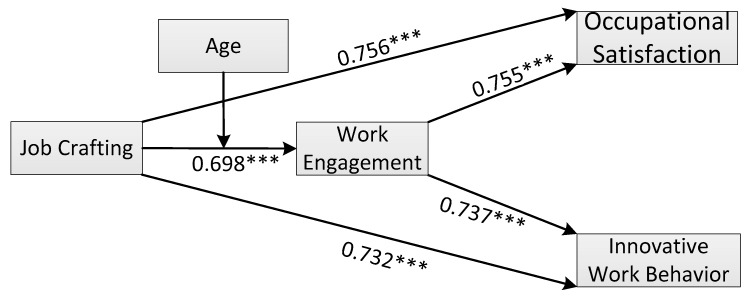
Path diagram and the standardized estimates of Model B. *** *p* < 0.01.

Table 2 shows that the partial mediation model (Model B; Table 2; χ^2^/df = 2.968, TLI = 0.920, IFI = 0.933, CFI = 0.933, and RMSEA = 0.08) fits better with the data than the non-mediation model (Model C; Table 2; χ^2^/df = 4.465, TLI = 0.860, IFI = 0.874, CFI = 0.873, and RMSEA = 0.106) and the full mediation model (Model A; Table 2; χ^2^/df = 3.575, TLI = 0.896, IFI = 0.909, CFI = 0.909, and RMSEA = 0.091). We therefore chose the partial mediation model to test our hypotheses; the standardized path estimates of this partial mediation model are presented in Figure 1.

As shown in Figure 1, we found that JC was significantly and positively related to occupational satisfaction (β = 0.756, *p* < 0.001) and IWB (β = 0.732, *p* < 0.001), supporting Hypotheses 1a and 1b, respectively. Next, JC was significantly and positively related to employee work engagement (β = 0.698, *p* < 0.001); thus, Hypothesis 2 was supported. Moreover, work engagement was significantly and positively related to occupational satisfaction (β = 0.755, *p* < 0.001) and IWB (β = 0.737, *p* < 0.001). As a result, Hypotheses 3a and 3b were also supported.

As far as the mediation Hypotheses 4a and 4b, we referred to Hayes and Preacher [47] and employed a bootstrapping analysis technique on the final model (Model B) to examine the indirect relationships. The bootstrapping technique is particularly suitable when inspecting the indirect relationships of mediation models [48]. According to MacKinnon et al. [46], the bootstrapping technique is considered more formal, powerful, and appropriate for examining the mediations than the Sobel’s [49] approach, because the bootstrapping approach does not consider the data set to be normally distributed. Further, the mediations or indirect relationships were deemed to be significant if the bias-corrected (BC) 95% confidence interval (CI) from 5000 bootstrap samples did not include zero (MacKinnon et al., 2002) [46].

The mediation findings suggest indirect relationships between JC and occupational satisfaction (bootstrap estimate = 0.2725; 95% CI = 0.1581, 0.4043) and between JC and IWB (bootstrap estimate = 0.2708; 95% CI = 0.1541, 0.3992). Hypotheses 4a and 4b received support, as work engagement not only directly affected occupational satisfaction and IWB, but also mediated the positive relationships of JC with occupational satisfaction and IWB.

**Figure 2:**
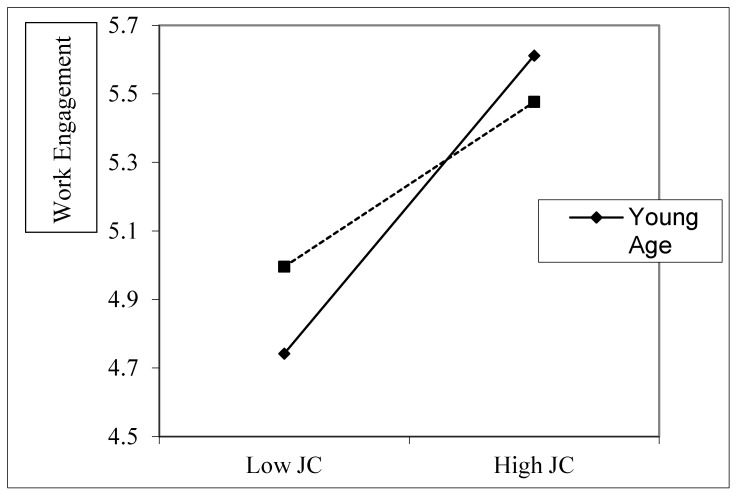
Moderation role of age on the mediation effect of work engagement on the JC (Job Crafting)–occupational satisfaction relationship.

As for the moderated mediation Hypotheses 5a and 5b, we proposed the moderation role of age on the mediation model. The results show that younger employees’ age strengthened the mediation model (JC→Work engagement→Occupational satisfaction; β = −0.0183, *p* < 0.01). Furthermore, a follow-up simple slope analysis demonstrated that the negative association between employees’ JC and work engagement was more pronounced among younger employees as compared to those who were older (Figure 2). Age moderated the mediating model, such that the mediating effect of work engagement on the JC–IWB relationship (β = −0.0187, CI = −0.0280, −0.0095). Hence, Hypothesis 5a and 5b were fully supported.

## 5. Discussion

This study examined the relationships of the expansive JCB with occupational satisfaction and IWB using a sample of manufacturing organizations in China. Our findings provide full support to our hypotheses: expansive JCB is positively related to occupational satisfaction, IWB, and work engagement, while work engagement is positively related to occupational satisfaction and IWB. Furthermore, work engagement partially mediated the JCB–occupational satisfaction and JCB–IWB relationships. Manufacturing workers’ age moderates the aforementioned mediating models, such that the mediating effect of work engagement on both the JC (Job Crafting)–occupational satisfaction and JCB–IWB relationships is stronger among younger employees. In sum, our study makes several contributions to the literature:

First and foremost, using the Chinese manufacturing industry, which is undergoing a radical transformation towards a high level of digitalization and automation and facing the threat of COVID-19, as our research setting, we discovered that the motivational mechanisms among the expansive JCB, occupational satisfaction, IWB, and work engagement could be activated by the increasingly complex job demands in a highly-uncertain, turbulent environment. Whereas relevant topics have mostly been investigated in Western contexts [24,50], our study that extends this line of career research to the Chinese context, thus enriching the literature derived from the Asian setting. More specifically, our findings highlight the vital role of the expansive JCB in fostering the employees’ subjective perceptions of their IWB and occupational satisfaction, which confirms that JCB also elicits positive attitudinal feedback among employees outside the Western cultural contexts.

Second, to our knowledge, despite a large body of research addressing the various associations among JCB, IWB, and work engagement [11,16,34,35,44,51], limited empirical studies have unpacked the psychological process of employees’ work engagement as a mediator to affect JCB–occupational satisfaction and ICB–IWB relationships from a cross-disciplinary angle integrating the perspectives of career and psychology research. We thus answer the calls of scholars to develop a deeper, direct connection (or even integration) of individual proactive behaviors to career-related outcomes [2,7,9].

Related to the point above, notwithstanding that the JD-R theory [4] has been commonly adopted in the domains of human resource management, psychology, and career [30] research, our findings still advocate the JD-R framework in a relatively novel way. More specifically, we demonstrated that facing the serious threat of job loss caused by the COVID-19 crisis, coupled with the application of ICTs and AI replacing human workers, manufacturing employees who actively craft their resources may exhibit more engagement and innovation at their work and feel more satisfied with their careers than those who undertake fewer self-initiated actions on their job changes. Moreover, younger employees seem to be more comfortable with and more capable of coping with the organizational changes caused by the COVID-19 pandemic.

In terms of practical implications, our results provide a valuable bridge connecting theory to practice. For instance, in the face of crucial innovation challenges driven by ICTs in the labor market, the expansive JCB can be used as an effective means for manufacturing firms to boost employees’ work engagement and IWB. Extending this line of thought, manufacturing firms may foster an appropriate organizational environment where fair evaluation and reward systems, effective operation processes, and relevant policies are all put in place, whereby their production workers can conduct expansive JCB more easily, and thereby to become more engaged and satisfied with their careers. It is thus imperative to better aligning employees’ expansive JC activities with the organization’s strategic goals so as to strengthen corporate competitive advantages, which to a certain extent allows employees’ JCB to become more meaningful and beneficial. In short, by giving employees opportunities to self-manage and craft their jobs, employers will be reciprocated with higher levels of productivity, and these employees will also be more likely to experience both personal and occupational growth. We thus offer valuable first-hand, insightful evidence about how manufacturing employees conducted JCB to re-orient their careers and to innovate in the face of the high unemployment situation.

## 6. Limitations and Future Research

The limitations of this study should be acknowledged. First, this study relies on employees’ self-reports, which makes the responses susceptible to biased estimates [52]. Future research may also pay attention to other ratings offered by more varieties of participants, such as direct colleagues, supervisors, or other stakeholders. Second, although we measured our dependent variables on a separate measurement time about three months later to control for common method variance (CMV) [41], CMV could still be present in the data and may have produced inflated correlations among our study variables. Third, we drew our sample for the survey from Chinese manufacturing firms, which may limit the generalizability of the findings to other work contexts and national cultures. Finally, our study indicates that work engagement can act as a critical mediator in JCB, IWB, and occupational satisfaction relationships [53]. Future research may seek to identify more alternative mediators. For instance, it would be rather interesting to take psychological capital into consideration, as it is a critical personal resource (i.e., hope, efficacy, resilience and optimism) that reflects a built-in creative tendency of individuals to develop multiple pathways for accomplishing their career goals and producing a variety of positive workplace outcomes [2,51,54].

## 7. Conclusions

In conclusion, echoing the JD-R framework, the present study provides encouraging empirical results that highlight the interrelatedness of JC strategies to strengthen employees’ occupational satisfaction and innovation performance, as well as the crucial mediating role of work engagement in such a critical time of COVID-19 and industrial transformation. We thus argue that our results enrich the existing body of knowledge by offering a better understanding of how individual employees leverage their job resources to cope with the constantly changing job demands in a complex, turbulent business landscape. Our research accentuates that, apart from work engagement, the expansive JCB can also largely promote employees’ growth in organizations and thereby should be given greater attention.

## Figures and Tables

**Table 1:** Means, standard deviations, inter-correlations, and reliabilities of the study variables.

Variable	1	2	3	4	5	6	7
1. Gender	-						
2. Age	0.120 *	-					
3. Experience	0.094	0.644 **	-				
4. Job crafting	−0.056	0.187	−0.070	**0.87**			
5. Work engagement	0.011	0.171 **	−0.069	0.658 **	**0.86**		
6. IWB	−0.058	0.130 *	−0.150 **	0.719 **	0.707 **	**0.88**	
7. Occupational satisfaction	0.033	0.149 **	−0.127	0.725 **	0.712 **	0.748 **	**0.86**
Mean	0.75	36.57	10.60	5.00	5.19	4.98	4.97
Standard deviation	0.436	8.378	8.820	0.665	0.650	0.661	0.599

Note: *N* = 311, * *p* < 0.05, ** *p* < 0.01. IWB = Innovative Work Behavior. The values of the square roots of AVE (Average Variance Extracted) were showed on diagonal in bold.

**Table 2:** Results of the structural equation modelling.

Model	χ^2^/df	TLI	IFI	CFI	RMSEA
Model A. Full mediation model	3.575	0.896	0.909	0.909	0.091
Model B. Partial mediation model	2.968	0.920	0.933	0.933	0.08
Model C. Non-mediation model	4.465	0.860	0.874	0.873	0.106

Note: *N* = 311. df = degrees of freedom; TLI = Tacker -Lewis Index; IFI = Incremental Fit Index; CFI= Comparative Fit Index; RMSEA= Root Mean Square Error of Approximation; Full mediation: did not include direct paths from JC (Job Crafting) to IWB (Innovative Work Behavior) and occupational satisfaction. Partial mediation: included direct paths from JC (Job Crafting) to IWB (Innovative Work Behavior) and career satisfaction. Non-mediation model.
